# Confirming a Drosophila Melanogaster Model of Huntingtin Aggregation

**DOI:** 10.1093/geroni/igab046.3678

**Published:** 2021-12-17

**Authors:** Akosua Biritwum, Simon Levy, Bess Frost, Atanu Duttaroy

**Affiliations:** 1 Howard University, Washington, District of Columbia, United States; 2 UT Health San Antonio, San Antonio, Texas, United States

## Abstract

For decades, doctors, psychologists, and psychiatrists alike have struggled to treat the symptomatic effects of Huntington’s disease. Huntington’s disease is an autosomal dominant brain disease that results in the deterioration of a person’s physical and mental state. Once a person inherits the disease, they end up dying from it more often than not. At present, there are 41,000 Americans with symptomatic Huntington’s disease, and 200,000 more are currently at-risk of inheriting the disease. Given its 50/50 chance of inheritance, there seems to be no end in sight to this degenerative ailment. My research study, however, will show that with a more robust approach, finding a cure for this disease is possible. Ultimately, the aim of this project was to test an already established model in Drosophila melanogaster regarding the “huntingtin” protein responsible for Huntington’s disease. This was achieved by first demonstrating that the flies which were modified to produce huntingtin could, in fact, produce the protein. Secondly, an experimental process was created to configure a system through which the amount of protein produced by each fly could be quantified. This quantification was vital in creating a baseline that would allow for the identification of potential therapeutic treatments in the future. In short, by establishing a quantifiable model for huntingtin, this study will pave the way to new insights on huntingtin aggregation and the identification of possible treatments for Huntington’s disease in the future.

